# Hyperkalemia or Not? A Diagnostic Pitfall in the Emergency Department

**DOI:** 10.5811/westjem.35286

**Published:** 2024-11-27

**Authors:** Frank-Peter Stephan, Florian N. Riede, Luca Ünlü, Gioele Capoferri, Tito Bosia, Axel Regeniter, Roland Bingisser, Christian H. Nickel

**Affiliations:** * University Hospital Basel, Emergency Department, Basel, Basel Stadt, Switzerland; † Bürgerspital Solothurn, Department of Cardiology, Solothurn, Solothurn, Switzerland; ‡ Kantonsspital Aarau, Department of Cardiology, Aarau, Aarau, Switzerland; § Karl Landsteiner University of Health Sciences, Krems an der Donau, Lower Austria, Austria; ∥ Le Centre Hospitalier Universitaire Vaudois (CHUV), Lausanne, Lausanne, Switzerland; ¶ MEDICA Medical Laboratories, Zürich, Zürich, Switzerland

## Abstract

**Introduction:**

Hyperkalemia, a potentially life-threatening electrolyte disturbance, is commonly encountered in the Emergency Department (ED). However, the frequency of factitious hyperkalemia, an artificially elevated potassium level in hyperkalemic ED patients, is unknown. This study aims to detect the rate of factitious hyperkalemia among patients with a potassium concentration of ≥5.0 mmol/l in an all-comer ED population.

**Methods:**

This retrospective, monocentric chart review analyzed data of 2,440 ED patients who presented with a potassium concentration of ≥5.0 mmol/L in their initial whole blood or plasma sample, who also underwent a repeat potassium measurement on the same day. Two groups were established based on potassium levels in the initial and repeat blood tests: 1) True hyperkalemia, characterized by consistently elevated potassium levels in both the initial and repeat samples; and 2) Factitious hyperkalemia, defined by an elevated initial potassium level while the repeat blood test showed a normal potassium level. A subset of factitious hyperkalemia was spurious hyperkalemia. In spurious hyperkalemia, the initial blood sample showed an elevated potassium level with evidence of hemolysis, but a repeat test revealed a normal potassium level without evidence of hemolysis.

**Results:**

Of the 2,440 patients, 1,576 (65%) had true hyperkalemia and 864 (35%) factitious hyperkalemia. Among the 864 patients with factitious hyperkalemia, 597 (69%) displayed hemolysis in their initial blood sample, indicating spurious hyperkalemia due to in-vitro hemolysis.

**Conclusion:**

These data show that about one third of all hyperkalemic blood samples drawn in the ED were due to factitious hyperkalemia. The leading cause of factitious hyperkalemia was spurious hyperkalemia due to in-vitro hemolysis.

Population Health Research Capsule What do we already know about this issue?

*Factitious hyperkalemia, an artificially elevated potassium level, should be ruled-out before treatment of hyperkalemia is initiated, as unnecessary treatment is not without risks.*

What was the research question?

*What is the rate of factitious hyperkalemia among patients with hyperkalemia in an all-comer ED population?*

What was the major finding of the study?

*Among 2,440 patients, factitious hyperkalemia occurred in 35% of hyperkalemic blood samples drawn in the ED.*

How does this improve population health?

*Factitious hyperkalemia is a diagnostic pitfall that can potentially lead to unnecessary treatment.*

## INTRODUCTION

Hyperkalemia is a potentially life-threatening and commonly encountered electrolyte disorder in the emergency department (ED). Elevated potassium concentrations are associated with increased mortality and, hence, require rapid initiation of potassium-lowering therapy.^1^ However, unnecessary treatment of hyperkalemia due to factitious hyperkalemia is not without risks.^2^ ^,^ ^3^ Surprisingly, the frequency of factitious hyperkalemia in hyperkalemic ED patients has not been determined. Therefore, our goal in this study was to determine the prevalence of factitious hyperkalemia among patients with a potassium concentration of ≥5.0 millimoles per liter (mmol/L) in an all-comer ED population (the primary outcome).

## METHODS

This retrospective, monocentric, chart-review study was approved by the ethics oversight committee (EKNZ identifier: 159/13). We evaluated 2,440 patients over a period of three years in whom a first whole blood or plasma sample showed a potassium concentration of ≥5.0 mmol/L at ED presentation and for whom a repeat (whole blood or plasma) lab test was ordered in the same patient (determined by a patient ID) on the same day by the treating physician. We adhered to 8 of 12 chart review criteria.^4^ Our protocol omits performance monitoring, hypothesis blinding, and interobserver reliability (IRR) tests, as abstractors were involved in designing the study, and IRR tests did not align with the study aim. Blood sampling and analysis was performed in the same manner for the initial and repeat blood test.

Blood was collected using peripheral venous catheters (Vasofix Safety Cannula, B. Braun AG, Melsungen, Germany) with a dual-use S-Monovette system allowing either aspiration- or vacuum-based blood sampling (Sarstedt AG, Nümbrecht, Germany). Plasma potassium concentration was analyzed using a Cobas 8000 analyzer (Roche Diagnostics, Basel, Switzerland), and whole blood samples were analyzed as part of a blood gas analysis with an ABL 800 analyzer (Radiometer, Copenhagen, Denmark). Strong agreement was found between the potassium measurements of the Roche Cobas 8000 analyzers.^5^ All scheduled calibrations for both devices were completed at 24-hour intervals in accordance with manufacturer requirements. The presence of hemolysis was either photometrically or visually assessed.

**Figure 1.**
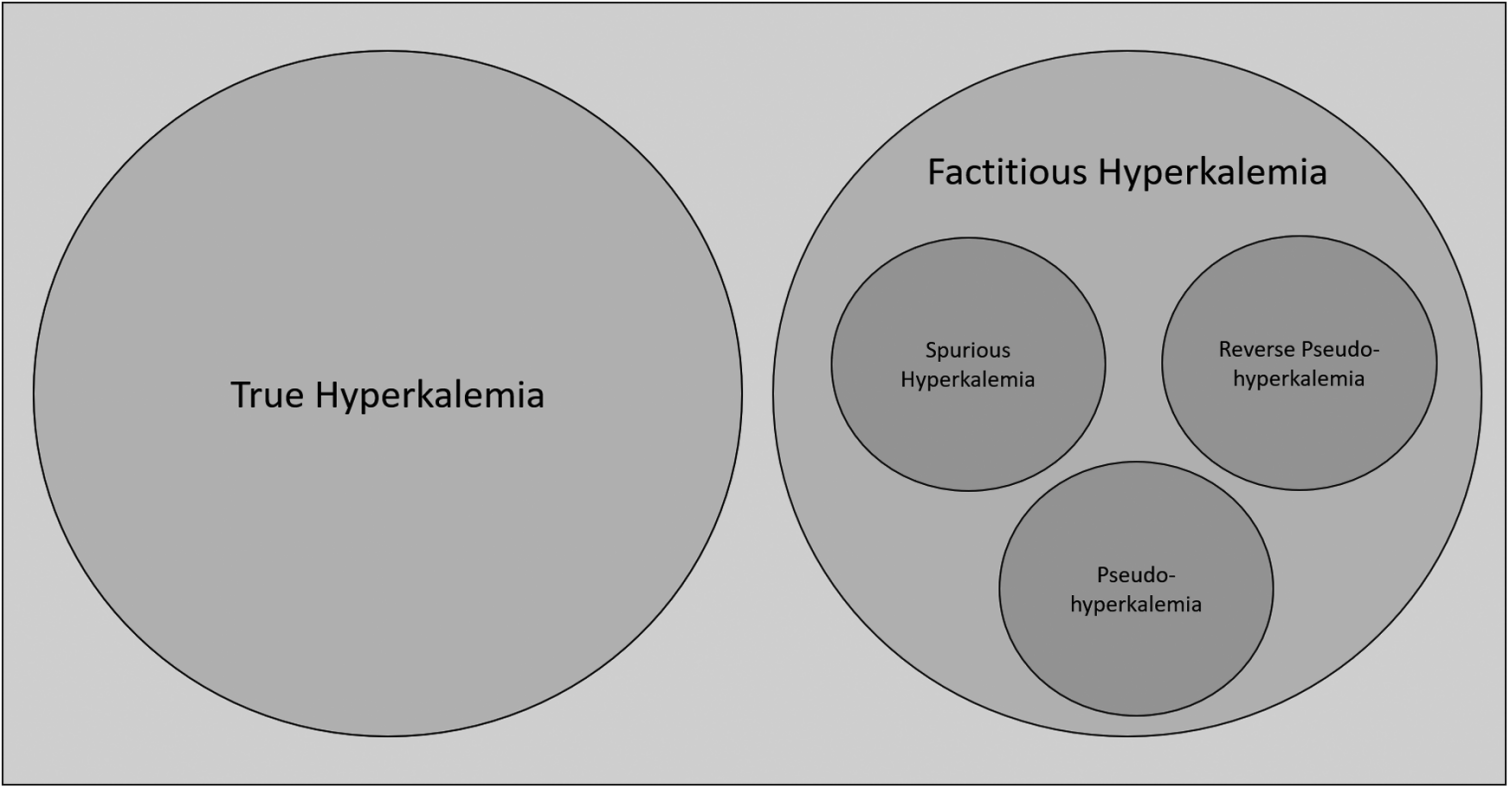
Venn diagram illustrating definitions of true hyperkalemia and factitious hyperkalemia. Factitious hyperkalemia is an umbrella term that encompasses various subcategories of falsely elevated potassium levels. These include reverse pseudohyperkalemia (in leukemic patients), pseudohyperkalemia (due to thrombocytosis or erythrocytosis), and spurious hyperkalemia (due to in-vitro hemolysis).

As there is no commonly accepted definition of factitious hyperkalemia, the terms pseudo-hyperkalemia, spurious hyperkalemia, and factitious hyperkalemia are often used interchangeably.^3^ ^,^ ^6^ ^–^ ^10^ To categorize our hyperkalemia findings, we established two distinct groups (see Figure 1): **true hyperkalemia**, characterized by consistently elevated blood potassium concentrations in both the initial and repeat blood test; and **factitious hyperkalemia**, which showed elevated blood potassium concentrations in the first sample while the repeat blood test showed a normal potassium level. Factitious hyperkalemia is an umbrella term for all sub-entities of falsely elevated potassium levels, as it does not differentiate between intrinsic (eg, hematologic disorders) and extrinsic causes (eg, blood sampling technique). Examples are reverse pseudohyperkalemia in leukemic patients or pseudohyperkalemia due to thrombocytosis or erythrocytosis. Another subset of factitious hyperkalemia is **spurious hyperkalemia**, in which an elevated potassium concentration with evidence of hemolysis was found in the first sample and a normal potassium in the repeat blood test. Spurious hyperkalemia is the result of hemolysis due to preanalytical errors during sampling or sample handling (ie, due to extrinsic causes).^6^

## RESULTS

**Figure 2.**
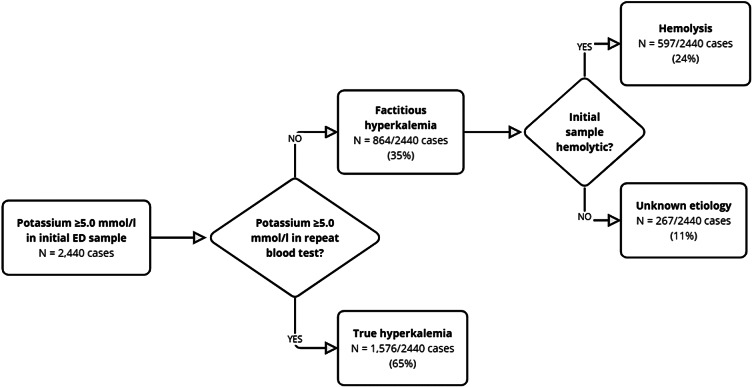
Proportions of patients that had true, factitious, and spurious hyperkalemia. Of 2,440 cases with a potassium of ≥5.0 millimoles per liter, 1,576 (65%) had persistent hyperkalemia in a same-day repeat blood test, (ie, they had true hyperkalemia); and 864 (35%) did not show hyperkalemia in a same-day repeat blood test; hence, these patients had factitious hyperkalemia. Of those 864 cases, 597 (69%) showed evidence of hemolysis in their initial blood test; thus, these patients had spurious hyperkalemia due to in-vitro hemolysis. In 267 of the 864 cases of factitious hyperkalemia (31%), no evidence of hemolysis was observed. The reason for the occurrence of factitious hyperkalemia in these cases remains unclear. *mmol/L*, millimoles per liter.

We included 2,440 patients with a potassium concentration of ≥5.0 mmol/L in the initial blood sample taken on the day of ED presentation. Patients with factitious hyperkalemia had a median age of 69 years, and 53% were female. Potassium levels of the factitious hyperkalemia group ranged from 5.0–29.9 mmol/L; median potassium concentration was 5.4 mmol/L. About one-third of the 864 hyperkalemia patients (35%) met the definition of factitious hyperkalemia, and 1,576 (65%) of the patients with hyperkalemia had true hyperkalemia (see Figure 2).

Of the 864 patients with factitious hyperkalemia, blood samples were rated as hemolytic in 597 cases (69%), indicating spurious hyperkalemia as the underlying etiology. In the remaining 267 of 864 cases (31%), the etiology of factitious hyperkalemia is unclear. Patients with true hyperkalemia had a median age of 75 years, and 41% were female. Potassium levels of the true hyperkalemia group ranged from 5.0–10.3 mmol/L; median potassium concentration was 5.4 mmol/L.

## DISCUSSION

This study shows that factitious hyperkalemia can be observed in about one-third of hyperkalemic blood samples drawn under real-life conditions in the ED. Spurious hyperkalemia due to preanalytical errors during blood sampling or transport leading to in-vitro hemolysis appears to be the main cause of falsely elevated potassium levels in this study. However, in critically ill patients many factors can influence one sample measurement (eg, transient acidosis or hypoinsulinemia with hypotension). Thus, it is unclear whether a first hyperkalemic measurement, which resolved in a second measurement, is factitious, or simply transient and quickly resolved. On the other hand, it is conceivable that there is true hyperkalemia in the cases that we classified as factitious hyperkalemia as there is also the phenomenon of pseudonormokalemia (ie, the repeat blood test is falsely low).^11^

In the process of analyzing the results of this study, we observed that the literature uses a variety of terms to describe factitious hyperkalemia.^3^ ^,^ ^6^ ^–^ ^10^ In cases of factitious hyperkalemia the underlying cause is often not considered, which might cause confusion. Surprisingly, this has not yet been acknowledged in hyperkalemia management guidelines.^3^ ^,^ ^10^ ^,^ ^12^ ^,^ ^13^ While our study shows that spurious hyperkalemia is a major contributor to factitious hyperkalemia in the ED, it also became apparent that the common assumption among clinicians that falsely elevated potassium levels are solely due to hemolysis resulting from inadequate blood-drawing techniques is false. There are numerous other significant underlying pathological processes that can lead to falsely elevated potassium levels (eg, thrombocytosis). While we cannot differentiate between all these underlying pathological processes in the ED, emergency physicians should be aware of the concepts of spurious, pseudo- and reverse pseudohyperkalemia, as potentially unnecessary treatment could be associated with risks.^2^ ^,^ ^3^

## CONCLUSION

This data shows that one-third of all hyperkalemic blood samples drawn in the ED are due to factitious hyperkalemia. The leading cause of factitious hyperkalemia is spurious hyperkalemia due to in-vitro hemolysis.
